# Occupational affiliation does not influence practical skills in cardiopulmonary resuscitation for in-hospital healthcare professionals

**DOI:** 10.1186/1757-7241-19-3

**Published:** 2011-01-14

**Authors:** Marie-Louise Södersved Källestedt, Anders Berglund, Ann-Britt Thoren, Johan Herlitz, Mats Enlund

**Affiliations:** 1 Uppsala University, Centre for Clinical Research, Västerås, Sweden; 2 Department of Medical Epidemiology and Biostatistics, Karolinska Institutet, Stockholm, Sweden; 3 School of Health and Caring Sciences, Linnaeus University, Växjö, Sweden; 4 University of Gothenburg, Sahlgrenska University Hospital, Gothenburg, Sweden

## Abstract

**Background:**

D-CPR (Defibrillator Cardiopulmonary Resuscitation) is a technique for optimal basic life support during cardiopulmonary resuscitation (CPR). Guidelines recommend that healthcare professionals can perform CPR with competence. How CPR training and provision is organized varies between hospitals, and it is our impression that in Sweden this has generally improved during the last 15-20 years. However, some hospitals still do not have any AED (Automated External Defibrillators). The aim was to investigate potential differences in practical skills between different healthcare professions before and after training in D-CPR.

**Methods:**

Seventy-four healthcare professionals were video recorded and evaluated for adherence to a modified Cardiff Score. A Laerdal Resusci Anne manikin in connection to PC Skill reporting System was used to evaluate CPR quality. A simulated CPR situation was accomplished during a 5-10 min scenario of ventricular fibrillation. Paired and unpaired statistical methods were used to examine differences within and between occupations with respect to the intervention.

**Results:**

There were no differences in skills among the different healthcare professions, except for compressions per minute. In total, the number of compression per minute and depth improved for all groups (*P *< 0.001). In total, 41% of the participants used AED before and 96% of the participants used AED after the intervention (*P *< 0.001). Before intervention, it took a median time of 120 seconds until the AED was used; after the intervention, it took 82 seconds.

**Conclusion:**

Nearly all healthcare professionals learned to use the AED. There were no differences in CPR skill performances among the different healthcare professionals.

## Introduction

Resuscitation guidelines have changed over the decades with the aim of increasing the chance of survival for a person with cardiac arrest [1]. All healthcare professionals should be able to perform cardiopulmonary resuscitation (CPR) with competence [2]. The Guidelines state that healthcare professionals should be able to start CPR within one minute, alert the hospital team within one minute, and use the Automated External Defibrillator (AED) within three minutes [3]. Training in D-CPR, include the use of an AED, which gives one defibrillation at a time, followed by 2 minutes of CPR [3,4].

Previous studies on high school students indicate that they can use an AED after education and practical training [5], and another study indicates that nurses can learn how to use an AED [6]. Not only the physicians or nurses are close to the patients. In addition, assistant nurses, physiotherapists and/or occupational therapists may be witnesses of a cardiac arrest. As far as we know there are no studies that have compared different healthcare professionals' practical skills. With this in mind, the present study was undertaken in order to investigate potential differences in practical D-CPR skills between different healthcare occupations before and after training according to the Swedish educational program, (slightly expanded version of the European Resuscitation D- program) [4].

## Materials and methods

The study was approved by the Regional Ethics Committee in Uppsala, Sweden (2006/201), and all participants gave their written consent after verbal and written information. The first test of the skills was accomplished in August 2006 to January 2007. The new European guidelines were presented at a National congress in November 2006 and the guidelines were in use at the studied hospital in May 2007. All data collected before education were evaluated according to guidelines 2000 [7] and all data collected after education were evaluated according to guidelines 2005 [4].

### Study participants

Participants were selected by their working managers at a hospital in Sweden, and with respect to their working schedules. The aim was to include 30 nurses, 30 physicians and 30 assistant nurses, physiotherapists and/or occupational therapists, who worked on ordinary wards and ICU, and emergency, medical, and surgical departments. The numbers of participants are illustrated in table 1.

**Table 1:** Demographic characteristics of the study participants and number of participants before and after education

Occupational title	Physician		Nurse		Assistant nurse*		Total	
	n	%	n	%	n	%	n	%
	
**Before education**								
Participants	28	-	31	-	29	-	88	-

**After education**								
Participants	23	82	26	84	25	86	74	84

**Gender**								
Male	16	70	3	12	3	12	22	30
Female	7	30	23	88	22	88	52	70

**Age before education**								
Median (range)	41	(28-71)	38	(25-57)	50	(21-62)	42.5	(21-72)

**Working experience**								
0-5 years	9	39	7	28	6	25	22	31
6-20 yeras	6	26	13	52	5	21	24	33
>20 years	8	35	5	20	13	54	26	36
Missing	0	0	1	0	1	0	2	0

* Assistant nurse includes the occupational titles assistant nurse, physiotherapists and occupational therapists.

A precision calculation estimate based on previous studies [8-12] was used as a guide for sample size calculation.

### Test protocol and data collection

In Sweden the use of AED is taught to healthcare professionals in a 4-hours course that also includes theory and practical training in basic CPR, use of oxygen and ventilation with mouth-to-mask technique, and use of suction devices for clearing of the airways. Study participants performed D-CPR on a manikin (baseline), attended the course, and performed D-CPR in the same set up 1-2 months after training (follow-up).

CPR training for the study participants was conducted during February 2007 to June 2007. The time from baseline evaluation until time for education varied between 1 to 5 months.

The follow-up evaluation was undertaken from March 2007 to July 2007. A period of 4-8 weeks elapsed between training and follow-up. Before the participants were asked to perform CPR on a manikin, they were asked to read a set of instructions:

"Imagine that you are somewhere in the hospital, and the person you are talking to suddenly becomes unconscious. You suspect a cardiac arrest. Perform and act as if you were at your own department. The instructor is in the room, but you cannot obtain any help from her, but you have to imagine her when you consider the safety of this situation. In the room, you can see an alarm switch; this is the only way to get help. You cannot go out of the room and ask for help. You decide by yourself if you want to perform mouth-to-mouth ventilation or use a ventilation mask, if you find it essential to perform ventilation. Do not move the manikin to the floor. The scenario takes about 5-10 minutes, this may appear a long time, but please continue to treat the person until the instructor tells you to stop. Thank you for your participation and good luck when the instructor gives you a sign to start".

Both at the baseline and follow-up evaluation, the scenario started with the manikin (Laerdal Skillmeter Resusci Anne, Laerdal Medical AS, Norway) in a hospital bed. A training AED (Laerdal Heartstart FR2, Laerdal Medical AS, Norway) and a ventilation/pocket mask was visible in the room, and could be used at the discretion of the participant. If participant choose to use the AED the first rhythm was ventricular fibrillation. The participants performed single rescuer CPR on the manikin and the entire scenario was video recorded (cf.: Appendix). The scenario was terminated after four minutes of compressions, measured from the first performed compression.

The Laerdal Skillmeter Resusci Anne includes a software program, a PC Skill reporting System for measuring vital functions during the simulated CPR situation. The measurements have a tolerance of ±15% for the variables compression depth and inflation volume. A rescue breath of minimum 250 ml was detected by the software as "ventilation", and a chest compression of at least 10 mm was detected as "compression". Correct compression depth was defined as 40-50 mm, and correct compression rate as 90-110/min. Correct ventilation volume was defined as 800-1200 ml before education (Guideline 2000) and 500-600 ml after education (Guideline 2005). The software program calculates a variable "compressions without error", which contains compressions with correct hand placement on the sternum, complete release and a compression depth of 40-50 mm.

Three experienced instructors evaluated the videotapes of the participants performing D-CPR in order to evaluate aspects of CPR not registered by the software program. The evaluation was accomplished according to the Cardiff test protocol [13]. To secure reliability each instructor received approximately 30 minutes of training in the use of the Cardiff test protocol. In addition, as a test, they separately evaluated one video-recorded participant performing D-CPR. Thereafter, the three instructors evaluated the same part together, to come to an understanding of the protocol. Then, two of the instructors evaluated all video recordings separately, and after 12 weeks they re-evaluated the video films. The re-evaluations were accomplished in order to estimate intra-observer variability and inter-observer variation. The third instructor served as a master control by evaluating a random sample of 10% of the recorded tapes in order to minimize the risk for bias in the evaluations.

### Statistics

Paired statistical methods were used for the analyses of before and after intervention within each profession and for all participants. Based on the assumptions for the tests, both parametric and nonparametric tests were considered. For parametric tests, the mean value with its standard deviation (SD) was calculated, and for nonparametric tests, the median with inter-quartile-range (IQR) was used. In order to compare the results between different professions, unpaired tests were applied. Inter-observer variability of video evaluations was assessed with Friedman's test. In the Cardiff protocol, in which the observed measure was in ordinal scale, the data were analyzed according to ordinal invariant measures for individual and group changes [14]. All tests were two-sided and statistical significance was considered as *P *< 0.05. All analyses were with the software program SAS version 9.2.

## Results

From the 90 participants, 88 (98%) took part in a standard 4-h training course, and 74 (82%) attended the follow-up.

The AED was used by 30 of the 74 (41%) participants before intervention and 71 (96%) of the participants after the intervention (*P *< 0.001) (Table 2). Before intervention, median time until the AED was used was 120 seconds (IQR 80-157 sec) and after intervention, the median time was 82 seconds (IQR 68-112.5 sec) (*P *< 0.001). The duration of the scenario was 2-7 minutes. By profession, the group of other healthcare professionals increased their use of AED most, (before 16%, after 96%, *P *< 0.001).

**Table 2:** Assessment of ventilations, chest compressions, and the use of the AED among all healthcare professionals before and after intervention

	Physicians			Nurses			**Other healthcare professionals**^**1**^			Total		
	**(n = 23)**			**(n = 26)**			**(n = 25)**			**(n = 74)**		

**Variables**	Before	After	*p*	Before	After	*p*	Before	After	*p*	Before	After	*p*
	
Number using the AED, n (%)	14 (61)	22 (97)	0.005	12 (46)	25 (96)	<0.001	4 (16)	24 (96)	<0.001	30 (41)	71 (96)	<0.001

**Ventilations**												
Ventilation volume ml, median (q1-q3)	321 (0-635)	670 (465-890)	0.006	735 (621-826)	656 (563-898)	n.s	441 (0-920)	726 (415-1081)	0.031	621 (0-815)	666 (444-928)	0.009
Correct ventilations with correct volume according to guidelines, %	3 (13.0)	3 (13.0)	n.s	5 (19.2)	4 (15.4)	n.s	8 (32.0)	1 (4.0)	0.020	16 (21.6)	8 10.8)	n.s

**Compressions**												
Number of compressions per minute, mean (sd)	48 (21)	53 (14)	0.031	47 (18)	64 (99)	<0.001	37 (17)	54 (15)	<0.001	44 (19)	57 (14)	<0.001
Compressions with no errors,* median (q1-q3)	24 (0-32)	39 (6-143)	0.012	27 (0-52)	76 (21-99)	0.009	1 (0-37)	24 (5-77)	0.024	18 (0-42)	55 (13-99)	<0.001
Compression depth mm mean (sd)	39 (10)	41 (8)	0.151	35 (9)	39 (7)	0.075	33 (13)	40 (7)	0.004	35 (11)	40 (7)	0.000
												

^1. ^Other healthcare professionals includes; assistant nurse, physiotherapists and occupational therapists.

q1-q3 = interquartile range (25% - 75%)

* Compressions with no errors includes correct placement of hands and adequate depth

sd = standard deviation

n = number of observations

### CPR characteristics

When comparing different healthcare professions after education, there were no differences in skills, except for compressions per minute. The median number of compressions was 53 per minute for physicians, 64 for nurses and 54 for the group of others. When comparing the number of compressions per minute between nurses and physicians there were a difference (*P *= 0.005), also when comparing this variable between nurses and the group of others (*P *= 0.007). Ventilation volume increased significantly from a median of 621 ml before intervention to 666 ml after the intervention (*P *= 0.009) (Table 2). Physicians increased their ventilation volume significantly from a median of 321 ml to 670 ml (*P *= 0.006), which was also evident in the group of other healthcare professionals (before median 441 ml, after median 726, *P *= 0.031). However, the latter group decreased the number of correct ventilations. In total, and stratified by occupation, the proportion of correct ventilations with correct volume according to guidelines was equal or decreased after the intervention (total before 22%, after 11%, *P *< 0.059).

### Video evaluation according to the Cardiff test protocol

When evaluating the videos according to Cardiff test protocol, the three observers evaluated all the recordings differently, except for the recording of checking/clearing the airway. The number of participants who did not open the airway increased after the intervention, but this difference was not statistically significant; before: 67%, after: 74% (*P *= 0.854). All other aspects of CPR, not registered by the software program, were not analyzed due to the unacceptable inter-observer variation.

## Discussion

### CPR characteristics

The main finding was that nearly all healthcare professionals learned to use the defibrillator and no major differences in CPR skills were detected among the different healthcare professions. One study investigating CPR skills among nurses found no differences in skills between nurses working in critical care units and nurses working on ordinary wards [15]. The present study added a new perspective by making comparisons between different healthcare professionals, whom all are expected to start CPR. The healthcare professionals, who participated before the intervention, but not after, did not differ in skills from those who completed the study.

The number of compressions per minute increased to 57/min after intervention. Correct compressions with adequate depth, according to the guidelines, was insufficient in all groups (before 5%, after 4%, p = 0.71). Compression depth increased after intervention, but needs to be deeper if guideline recommendations are to be followed, as in a study by Curry et al. [16].

During the study period, the Guideline recommendations changed. Before intervention, the correct ventilation volume was 800-1200 ml (guideline 2000), whereas in the new Guideline (Guideline 2005), it was 500-600 ml. Consequently, Guideline 2000 [17] was used before intervention and Guideline 2005 [18] was used after the intervention. For all healthcare professionals, the median ventilation volume was 621 ml before and 666 ml after intervention. Thus, the Guideline recommendation in ventilation was not attained neither before, nor after intervention. The ventilation results were consistent with other studies, indicating CPR skills are poorly received [19]. One study determined that 50% of ventilation attempts are unsuccessful due to airway obstruction [20,21], which did not corroborated with the findings in this study.

### Video evaluation according to the Cardiff test protocol

It is difficult to evaluate practical skills in different studies, as the authors choose different evaluation methods [13]. The purpose of using the Cardiff test was to enable generalization and comparison of the findings with other studies. The authors of the Cardiff test protocol state reliability is less acceptable in variables such as checking for responsiveness, initial checking/clearing of the airway, and checking for signs of circulation. In this study, the instructors evaluated the videotapes in different ways, indicated by large inter-observer differences; therefore, the results could not be interpreted. Future studies are needed to address this in more detail.

### General discussion

CPR skills did not differ among healthcare professionals. However, the skills did not attain guideline levels in any professional group. Unfortunately, these results concurred with other studies, indicating limited improvement in resuscitation skills [22]. Practical skills need to be tested, and a written evaluation test only is not sufficient, as healthcare professionals appear to perform better in written tests than in skills tests [23]. Since physicians have a high level of theoretical knowledge already before passing a CPR course, we have speculated that CPR courses might need to be adapted to healthcare professionals' occupation. In general, physicians do have less time for education and repetition training. If the course were adjusted for their practical training needs, maybe more physicians would come for repetition training? Assistant nurses, physiotherapists and/or occupational therapists have less theoretical knowledge from the beginning, indicating their need for more regular theoretical and practical training. All professionals need at least one annual CPR course.

Even if the ventilation and compression skills were hard to perform according to guidelines, the majority learned to use the AED. Other studies conclude high school students can use an AED after education and practical training [5], and another study indicates nurses can learn how to use an AED [6]. The guidelines offer a uniform way of teaching CPR [3], and the 2005 guideline simplifies the resuscitation techniques [4]. Nevertheless, one study [24] indicates instructors do not teach in a standardized way and that poor CPR skills among participants may reflect the instructor. Consequently, we cannot exclude that this may be the case in the present study, although guideline adherence was stressed to the instructors.

The main purpose of this study was to investigate potential differences in practical CPR skills between different healthcare professionals. If any healthcare professional are less skilled, it would affect the outcome negatively for the patient. This study indicates that it does not matter which occupational healthcare professional who perform CPR.

### Strengths and limitations

Simulation differs from a real situation and CPR manikins need to have realistic body structure [25]. Although the study manikin has a realistic body structure, the authenticity of the scenario can still be questioned [26].

Even if the healthcare professionals were selected by working managers with respect to working schedules, resulting in quasi-randomization, the risk for selection bias cannot be excluded. Although different healthcare professions were included in the study, occupational group could hamper the results due to few participants in stratified analyses.

There was no specific time frame recommended for the interval for assessment of inter-observer variability in the evaluation of videotapes [13]; therefore, 12 weeks was chosen for practical reasons. The follow-up of the healthcare professionals was 4-8 weeks after education: this period was not based upon scientific evidence.

The change in correct ventilation volume in the 2005 guidelines may have affected the results. The participants may have a memory from the test before education of giving insufficiently low ventilation volumes. Despite the information and the training in reduced target volume, according to the new guideline, they may have been unable to adapt to a lower volume.

The data program used for evaluation has some uncertainty with a tolerance of ±15%. The program was used to get more exact information about the practical skills. As an example, it is hard for a person to count the compression rate by themselves, whereas the program gives a uniform way of evaluating practical skills. This makes it possible to compare results from different studies.

## Conclusion

A positive outcome was determined concerning the ability of learning to use an AED by all groups of healthcare professionals. There were no major differences in skills between the different healthcare professionals. However, the results for important skills, such as different aspects of chest compressions and ventilation, were poor, indicating more efforts is required in repetitive training of CPR skills for all healthcare professional categories.

## List of abbreviations

AED: automated external defibrillator; BLS: basic life support; CPR: cardiopulmonary resuscitation; DVD: digital versatile disc; D-CPR: Defibrillator Cardiopulmonary Resuscitation; ERC: European resuscitation council; ICU: intensive care unit; IQR: inter-quartile-range; SD: standard deviation; S-CPR: Hospital Cardiopulmonary Resuscitation including oxygen and equipment for vacuum suction.

## Competing interests

The authors declare that they have no competing interests.

## Authors' contributions

MLSK participated in the design and planning of the study, collected the data, participated in the statistical analysis, wrote the manuscript draft, and co-ordinated the subsequent versions of the manuscript. ME participated in the design and planning of the study and was involved in drafting the manuscript and the statistical analysis. JH revised the study manuscript and made important additions. AB performed the statistical analysis and revised the manuscript. ABT participated in the evaluations of the video-films and revised the manuscript. All authors read and approved the final manuscript.

## Appendix

These are the expected actions during the scenario:

- Check responsiveness

- Initial airway opening

- Initial breathing check

- Alarm/Phone

- Switch on the AED, initial rhythm VF

- Attaches the electrodes

- Visual and verbal hands-off checks during AED analysis

- Perform CPR, use ventilation mask or mouth to mouth ventilation

- Interrupt CPR (when AED tell to do so after 2 minutes)

- Visual and verbal hands-off checks during AED analysis

- Perform CPR, use ventilation mask or mouth to mouth ventilation during 2 minutes
